# Gender-moderated effects of academic self-concept on achievement, motivation, performance, and self-efficacy: A systematic review

**DOI:** 10.3389/fpsyg.2023.1136141

**Published:** 2023-03-28

**Authors:** Leihong Wang, Zhonggen Yu

**Affiliations:** ^1^Department of Linguistics, Beijing Language and Culture University, Beijing, China; ^2^Faculty of Foreign Studies, Beijing Language and Culture University, Beijing, China

**Keywords:** academic self-concept, achievement, motivation, performance, self-efficacy, gender differences

## Abstract

Although academic self-concept plays a crucial role in promoting students' education, there is a paucity of studies simultaneously exploring the gender-moderated effects of academic self-concept. This study aimed to explore gender-moderated effects of academic self-concept on achievement, motivation, performance, and self-efficacy. With Preferred Reporting Items for Systematic Reviews and Meta-Analyses Protocol (PRISMA-P) and STARLITE criteria, this study screened and assessed the retrieved literature, finally including 53 studies. It was concluded that academic self-concept exerted a positive influence on improving achievement, enhancing motivation, ameliorating performance, and boosting self-efficacy. It should also be noted that interrelations between academic self-concept and other educational constructs may be much more complicated than expected since gender disparities may moderate the effects of academic self-concept. Gender discrepancies in academic self-concept could account for the gap between male students and female students in subject-specific achievement, motivation, performance, and self-efficacy, especially in STEM courses. Teaching interventions and educational policies should be taken to enhance female students' STEM courses self-concept. Future studies should promote educational equality, highlight academic self-concept of special groups, and enhance academic self-concept in online learning.

**Systematic review registration:**

https://osf.io/uxjnv/?view_only=b10db44d34154d96a361c159ca15a5b5.

## Introduction

Academic self-concept has been widely acknowledged as an indispensable goal in the educational enterprise (Jansen et al., 2020). Academically successful students are generally characteristic of positive academic self-concept, strong motivation, and desirable academic behaviors (Burger and Naude, 2020). As a matter of fact, academic self-concept is not only a desirable educational goal but also a significant means of boosting students' academic outcomes and improving educational behaviors (Marsh et al., 2014). The COVID-19 pandemic compelled teachers and students to switch from offline learning to online learning, which exerted a negative effect on students' academic self-concept (Emmerichs et al., 2021). Fortunately, it is reported that high academic self-concept and motivation may be conducive to enhancing students' performance and achievement emotions, especially in adverse circumstances (Paechter et al., 2022).

It has been demonstrated that gender differences could make a difference to students' formation and development of academic self-concept (Postigo et al., 2022). The stronger the gender stereotypes in educational settings, the more pronounced the gender-moderated effects on academic self-concept (Koivuhovi et al., 2019). To some extent, gender disparities in academic self-concept accorded closely with gender stereotypes (Ertl et al., 2017). To put it simply, male students maintaining higher math self-concept tended to excel at mathematics. On the contrary, female students holding positive verbal self-concept were more likely to be proficient in languages (Espinoza and Taut, 2020). Moreover, in terms of academic self-concept, there existed larger gender differences in gifted students than average-ability students (Preckel et al., 2008).

Some reviews have been conducted to explore gender-moderated effects of academic self-concept on educational constructs. A moderating effect of gender has been found in social comparison in the classroom, with gender differences negatively affecting female students' expectations of success and self-concept (Dijkstra et al., 2008). Gender stereotypes could exert negative influences on female students' mathematics and science self-concept, causing female students' under-participation in Olympiads (Steegh et al., 2019). Compared with boys, girls with lower self-efficacy suffered from higher math anxiety (Kaur et al., 2022). The class composition may make a difference to female students' academic self-concept, with the single-sex class being beneficial to female students' science-related self-concept (Belfi et al., 2012).

Despite ever-increasing attention has been paid to academic self-concept and gender differences, there is a paucity of studies simultaneously synthesizing the gender-moderated effects of academic self-concept on a couple of educational constructs (see Table 1). Given the academic self-concept's great significance in boosting educational outcomes, it is indispensable to make a systematic review regarding gender-moderated effects of academic self-concept on achievement, motivation, performance, and self-efficacy. This study firstly makes a brief review of the above-mentioned constructs and gender differences separately, then strives to examine the gender-moderated effects of academic self-concept on achievement, motivation, performance, and self-efficacy. Finally, implications for future research are made to improve educational outcomes and strive for education equality.

**Table 1:** A comparison between previous studies and the current study.

**N**	**References**	**Data range**	**Academic topics**					
			**Self-concept**	**Achievement**	**Motivation**	**Performance**	**Self-efficacy**	**Gender**
1	Dijkstra et al. (2008)	1954–2007	√	✗	✗	✗	✗	√
2	Steegh et al. (2019)	2008–2017	√	✗	✗	√	✗	√
3	Kaur et al. (2022)	1990–2020	✗	✗	✗	✗	√	√
4	Belfi et al. (2012)	2000–2011	√	✗	✗	✗	✗	√
5	Current study	2008–2022	√	√	√	√	√	√

## Literature review

### Academic self-concept

It was necessary to clarify the definition and classification of academic self-concept. Academic self-concept was regarded as a facet of self-concept (Jónsdóttir and Blöndal, 2022). Different from physical, social, and emotional self-concept, academic self-concept was specifically bound up with academic outcomes (Vu et al., 2022). Therefore, academic self-concept could be defined as students' self-perception of their current academic competence (Marsh and Martin, 2011; Paechter et al., 2022). Academic self-concept was widely acknowledged as both multidimensional and hierarchical construct (Beaudrie, 2018). Academic self-concept could be subject-related (Shavelson et al., 1976; Arens et al., 2016), such as English self-concept and history self-concept, or skill-related (Arens and Jansen, 2016), such as reading self-concept and writing self-concept. Besides, academic self-concept could also be roughly divided into verbal self-concept and mathematics self-concept (Vu et al., 2022).

Academic self-concept was formed in the interplay of social, dimensional, and temporal comparison (Wolff and Möller, 2022). The internal/external frame of reference model demonstrated that academic self-concept was significantly interrelated with achievement within and across domains (Arens and Preckel, 2018). For instance, if student A with lower verbal achievement compared with student B with higher verbal achievement, then student A may be stuck with a lower self-concept (Kavanagh, 2020). Meanwhile, if one student excelled in verbal affairs but underperforms in mathematics, then this student may undergo a lower mathematic self-concept (Lohbeck and Möller, 2017). Moreover, big-fish-little-pond effect (henceforth “BFLPE”) claimed that students usually suffered from lower academic self-concept, in company with higher-achieving classmates than lower-achieving classmates (Aguillon et al., 2020).

### Achievement

Achievement could be generally defined as positive long-term outcomes derived from academic learning (Tomás et al., 2020). Achievement was assumed to be based on cognitive ability (Chen et al., 2012). Multiple factors may exert an influence on achievement, such as individual characteristics, teachers' instructions, expectancy (Friedrich et al., 2015), and academic environment. According to expectancy-value theory, actual academic achievement could be prominently predicted by expectancy for success (Cambria et al., 2017). Several ways were available to measure students' achievement, such as school grades, achievement test scores and, grade point average (Arens et al., 2022).

Abundant studies have been conducted to explore the reciprocal association between academic self-concept and achievement (Wu et al., 2021). Nevertheless, the interrelation between academic self-concept and achievement may be more complex than supposed to be (Keller et al., 2021). Besides, whether gender differences could exert an influence on the interplay between academic self-concept and achievement remained controversial (Cambria et al., 2017). Given the inconsistent findings, it is worthwhile to scrutinize the literature and account for the intricate interrelations between academic self-concept and achievement.

### Motivation

Motivation was regarded as a decisive factor influencing students' learning process and academic success (Fadda et al., 2022). Motivation may act as an indispensable part of boosting educational outcomes, such as perseverance and performance (Wigfield and Eccles, 2000). Motivation was generally composed of intrinsic and extrinsic motivation (Paechter et al., 2022). Intrinsic motivation may involve inherent satisfaction derived from engaging in or accomplishing an academic task (Brisson et al., 2017). On the contrary, extrinsic motivation may relate to the aim of achieving some educational outcomes (Brisson et al., 2017), such as achievement-oriented behaviors or performance.

The available researches have substantially recognized the significance of motivation, but not enough attention has been paid to gender differences in academic self-concept on motivation. Although both boys and girls suffered from motivational decreases (Scherrer and Preckel, 2019), gender-moderated effects of academic self-concept on motivation may vary from boys to girls (Wirthwein et al., 2020). Therefore, there is a need to review the effects of academic self-concept on motivation from the perspective of gender differences.

### Performance

As an academic indicator, performance was a prominent reflection of academic self-concept (Gorges et al., 2018). Moreover, performance was an important educational outcome in students' academic careers (Burger and Naudé, 2019). Performance could be deemed as how students handled with their academic tasks with instructions from teachers (Wigfield et al., 2020). As often is the case, performance was measured by means of school grades, grade point average, or standardized achievement tests (Steinmayr et al., 2018). Among negative performances, there was an increasing trend of test anxiety in school-aged children (Raymo et al., 2019).

Considerable researches have demonstrated that performance was positively related with academic self-concept (Colmar et al., 2019). However, little research has touched on whether academic self-concept could directly predict performance and whether gender identity could make a difference to students' performance (Aguillon et al., 2020). Therefore, there is an urgent need to review the interrelation between academic self-concept and performance in terms of gender differences, alleviating students' test anxiety and fostering students' academic buoyancy.

### Self-efficacy

Self-efficacy was an integral determinant of academic decisions and outcomes (Tomás et al., 2020). Self-efficacy could be defined as students' self-perceived confidence to accomplish educational tasks or achieve academic goals (Arens et al., 2022). Self-efficacy also referred to one's ability to effectively control academic activities and foster capabilities (Wan et al., 2021). Academic self-efficacy was of great significance in education enterprise (Arens et al., 2022) and it could make a great difference to students' thoughts and behavior. Strong self-efficacy usually made students courageous to meet academic challenges while weak self-efficacy made students fearful of engaging in academic activities (Scherrer and Preckel, 2019).

Although extensive researches have been conducted on the significance of self-efficacy (Arens et al., 2022), inconsistent findings remained to be systematically reviewed, such as whether academic self-concept and self-efficacy could mutually influence each other (Scherer, 2013). Moreover, gender differences should be taken into consideration as regards the effects of academic self-concept on self-efficacy. Clarifying gender differences in self-efficacy was conducive to promoting female's representation in STEM careers, ranging from science, technology, engineering to mathematics (Aguillon et al., 2020).

### Gender differences

Gender was an influential variable in educational settings (Cooper et al., 2018). Gender may influence students' interest in science lessons (Cheung, 2018). Gender stereotypes referred to a generalization of social expectation and habitual attribution in terms of a particular gender's abilities (Ertl et al., 2017; Savolainen et al., 2018). It was assumed that there were differences in academic abilities and characteristics among male and female students, accounting for their discrepant behaviors (Bieg et al., 2015), such as girls may hold higher verbal self-concept while boys may excel in math self-concept (Espinoza and Taut, 2020) and these gender discrepancies may become increasingly prominent over time (Valls, 2022). Therefore, the influence of gender differences in education should not be overlooked.

Although some studies have recognized gender differences in educational settings, scarce research explored the gender-moderated effects of academic self-concept on achievement, motivation, performance, and self-efficacy. According to a longitudinal study, moderation effects of gender could be found between expectancy-value belief and courses participation, performance, and preparation among Hispanic youth (Safavian, 2019). Once math-related gender stereotypes were widely acknowledged by the whole class, then female students' math self-concept would be negatively affected (Wolff, 2021). Gender differences seemed to constantly make a difference to students' academic behaviors, particularly during the transition from elementary school to middle school (Savolainen et al., 2018).

### Objectives and research questions

This study intends to systematically review whether academic self-concept could exert an influence on achievement, motivation, performance, and self-efficacy. The study focuses on the following five questions: (1) Can academic self-concept influence achievement? (2) Can academic self-concept influence motivation? (3) Can academic self-concept influence performance? (4) Can academic self-concept influence self-efficacy? (5) Can gender differences moderate the effects of academic self-concept on achievement, motivation, performance, and self-efficacy?

## Research methods

### Research design

**Figure 1:**
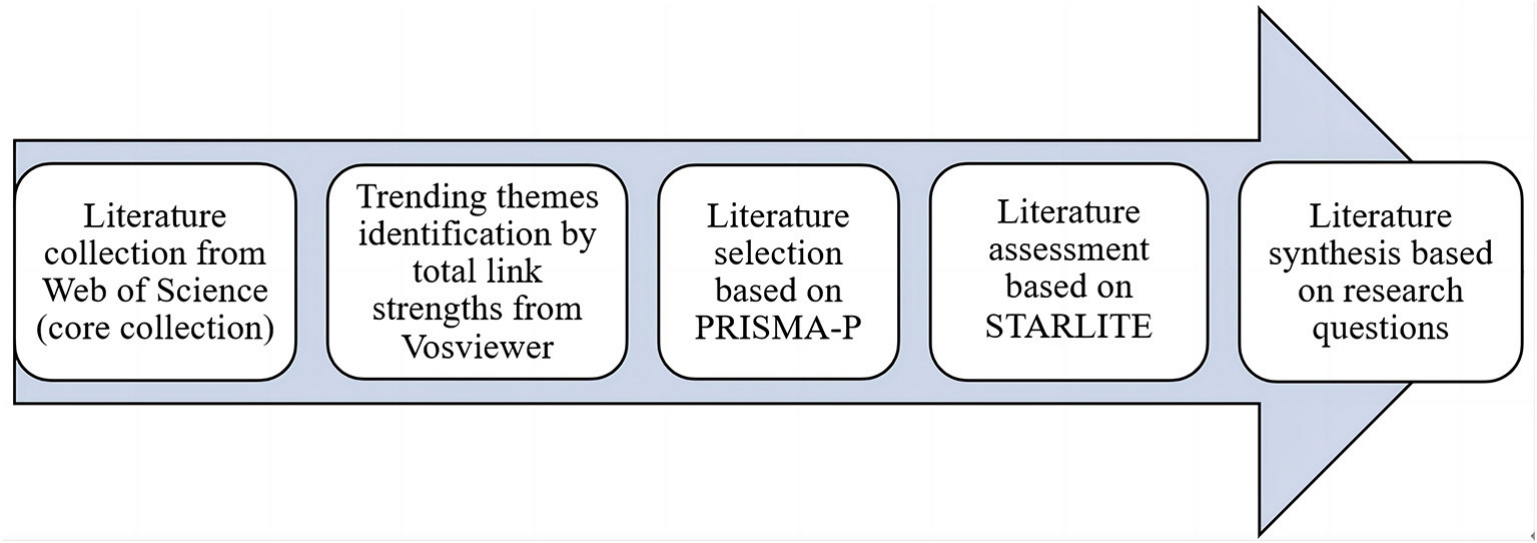
Steps of literature collection and selection.

This study adopted rapid evidence assessment to review the previous literature systematically. Figure 1 illustrated how this study dealt with literature review on academic self-concept. Firstly, this review retrieved all the relevant literature from Web of Science (core collection). Secondly, this review scrutinized the much-debated themes and then put forward the corresponding research questions by virtue of clustering and link strengths supported by VOSviewer. Thirdly, this review screened the literature under the guideline of Preferred Reporting Items for Systematic Review and Meta-analysis (PRISMA) (PRISMA-P Group et al., 2015). Fourthly, this review assessed literature quality with STRALITE (Booth, 2006). Lastly, this review explored the gender-moderated effects of academic self-concept in terms of achievement, motivation, performance, and self-efficacy.

### Research corpus

This study collected relevant literature from Web of Science (core collection) on October 16, 2022. Web of Science was known as a digital database with abundant high-quality academic articles. For the sake of comprehensiveness, this study retrieved all the data from Web of Science sub-databases accessible to the school library. The literature retrieved, ranging from January 2008 to October 2022, amounted to 1128 results, by keying in “academic self-concept” (topic) and student^*^ OR learn^*^ OR educat^*^ (topic) through Boolean logic. Given that some articles belong to irrelevant fields, such as psychiatry and management, etc., this study narrowed down the scope to only focus on education and educational research, resulting in 763 results.

**Figure 2:**
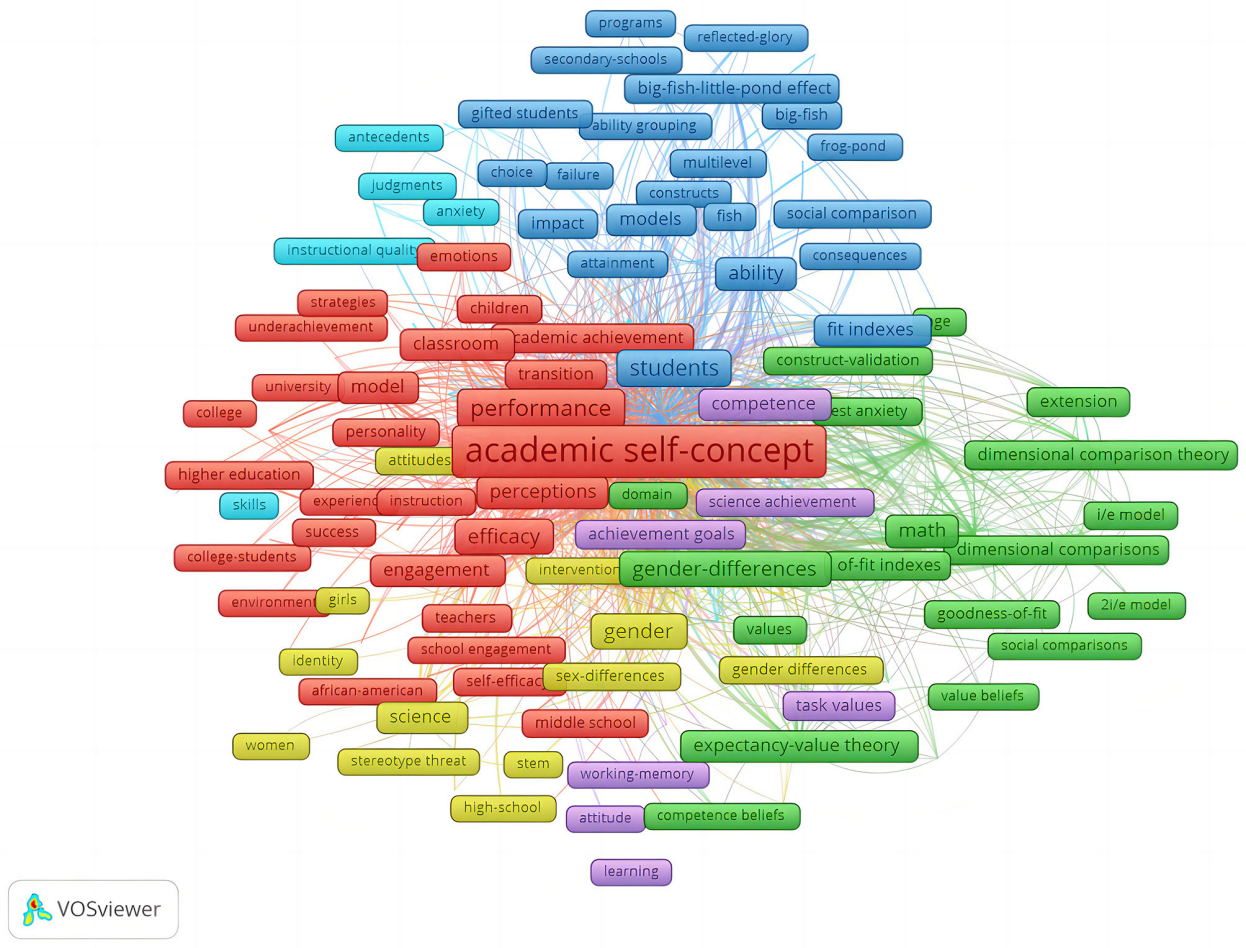
The bibliographic network.

To accurately identify trending themes, this study employed VOSviewer to visualize the bibliographic network. Firstly, this study imported the bibliographic data downloaded from Web of Science. Secondly, this study chose the reasonable type of analysis, unit of analysis, and counting method. Taking trending themes into consideration, the type of co-occurrence plus all keywords was more suitable. This study assumed each co-occurrence with the same weight, and adopted full counting as the counting method. Thirdly, as for the threshold, this bibliographic network admitted a keyword occurring at least eight times. This resulting in successfully identifying 209 items from the 2612 keyword searched, which are able to meet the base line requirement. The network visualization was shown in Figure 2. As depicted by the colors in Figure 2, the bibliographic network consisted of 6 clusters including a total of 209 keywords. Cluster 1 represented 70 items, e.g., academic achievement, academic motivation, academic performance, academic self-concept, and efficacy. Cluster 2 represented 40 items, e.g., academic self-concept, academic achievement, competence belief, dimensional comparison, expectancy-value theory. Cluster 3 represented 36 items, e.g., ability, ability grouping, achievement, attainment, aspirations, big-fish-little-pond effect, and social comparison. Cluster 4 represented 27 items, e.g., attitudes, beliefs, gender differences, intervention, stereotype threat, and resilience. Cluster 5 represented 20 items, e.g., achievement goals, attitude, competence, goal orientation, intrinsic motivation, and math anxiety. Cluster 6 represented 16 items, e.g., achievement motivations, antecedents, anxiety, etc.

**Figure 3:**
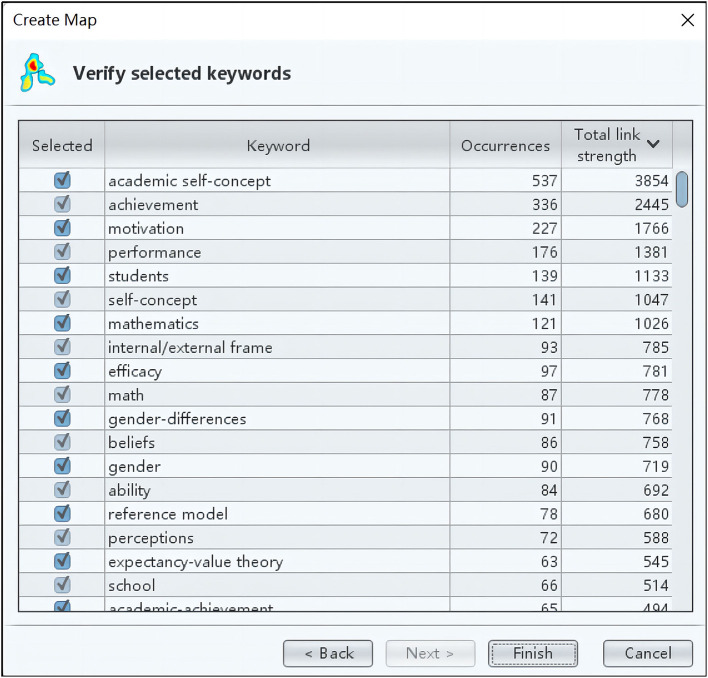
Total link strengths.

Apart from the cluster analysis, VOSviewer provided the list of keywords based on total link strengths. As was clearly shown in Figure 3, academic self-concept has the strongest link strength (*N* = 3,854), ranked first in the list. In addition, the link strengths of achievement (*N* = 2,445), motivation (*N* = 1,766), and performance (*N* = 1,381) are also highly ranked. But what should not be ignored is that the item self-efficacy with the total link strength (*N* = 781) is ranked top 10. Judged from the clusters and total link strengths, it is demonstrated that self-concept, achievement, motivation, performance, and self-efficacy are trending themes.

### Literature selection

**Figure 4:**
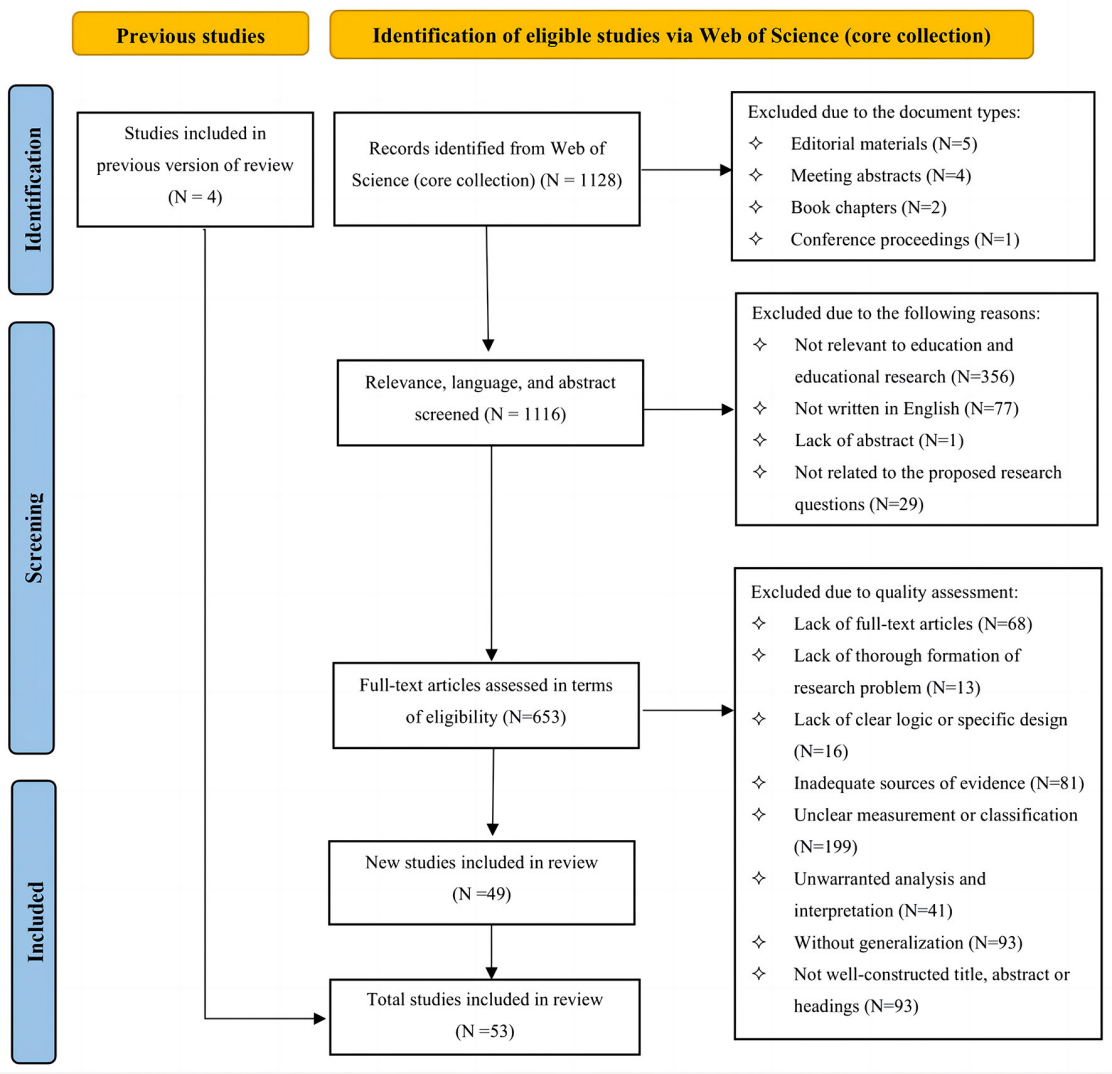
The PRISMA flow diagram of literature selection.

The researchers screened the previously retrieved literature by means of Preferred Reporting Items for Systematic Review and Meta-analysis Protocol (PRISMA-P). As shown in Figure 4, a total of 1,128 records are obtained from Web of Science (core collection). In terms of document types, a total of 12 records are excluded, such as editorial materials (*N* = 5), meeting abstracts (*N* = 4), book chapters (*N* = 2), and conference proceedings (*N* = 1). In terms of relevance, language, and abstract, 463 more records are excluded, including records beyond the field of education and educational research (*N* = 356), records not written in English (*N* = 77), records without abstract (*N* = 1), and records irrelevant to the proposed research questions (*N* = 29).

After 2 rounds of screening, a total of 653 full-text articles needed to be assessed in terms of eligibility. Thereinto, the full text of 68 records was not accessible. Apart from that, the rest literature was screened based on the AERA reporting standards. The literature was excluded due to the following reasons, lack of thorough formation of the research problem (*N* = 13), lack of clear logic or specific design (*N* = 16), inadequate sources of evidence (*N* = 81), unclear measurement or classification (*N* = 199), unwarranted analysis and interpretation (*N* = 41), without generalization (*N* = 93), and not well-constructed title, abstract or headings (*N* = 93). 5 previous literature reviews were added. Finally, a total of 53 studies were covered for this systematic review.

### Literature assessment

To conduct the study effectively, the retrieved articles should be both comprehensive and topic-oriented. STARLITE may be employed as a practical method to assess the quality of literature. The mnemonic STARLITE stands for the following eight standards, sampling strategy, type of study, approaches, range of years, limits, inclusion and exclusions, terms used, and electronic sources respectively (Booth, 2006). Table 2 is designed to demonstrate whether all the above standards were satisfied or not. According to the illustrations in Table 2, the present study generally satisfies the standards for systematic reviews with refined results based on STARLITE. Moreover, two raters undertook literature assessment (“agree” = 2, “partially agree” = 1, “disagree” = 0), and the inter-rater concordance (*k* = 0.941) indicated that the included literature was of good quality.

**Table 2:** Self-check based on STARLITE.

**Check**	**Elements**	**Detailed explanation**
✓	S: sampling strategy	Comprehensive sampling enabled all the relevant literature accessible to the study; Selective sampling made the retained literature within education and educational research field.
✓	T: types of studies	Both macro and micro researches were included, varying from qualitative study, quantitative study to mixed study.
✓	A: approaches	Hand-searching was incorporated into electronic subject searches by means of flexible adjustment of Boolean logic search. For example, the terms “academic self-concept”, “educat^*^”, “learn^*^”, “student^*^”, “achievement^*^”, “motivation”, “performance”, and “efficacy” were chosen as the topic.
✓	R: range of years	The literature retrieved is from January 2008 to October 2022; Figure 5 shows the number of publications on academic self-concept.
✓	L: limits	This study set limits on four aspects, time, document types, the language in which the articles were written. Specifically, the articles, except book chapters, etc., written in English, published from 2008 to 2022, within education and educational research domain were included.
✓	I: inclusion and exclusion	Some literature were included while others were excluded based on the following criteria, whether the publication date ranged from 2008 to 2022; whether the literature was within education and educational research scope; whether the literature was relevant to academic self-concept, achievement, motivation, performance, self-efficacy and gender differences; whether the literature offered adequate samples or statistics; whether the literature was rigidly designed; whether the results or conclusions were clear and convincing.
✓	T: terms used	The terms, such as “academic self-concept”, “achievement”, “motivation”, “performance”, “self-efficacy”, and “gender”, were fully present.
✓	E: electronic sources	All the accessible 6 sub-databases from Web of Science (core collection) were covered.

### Literature synthesis

This study adopted the comparative thematic approach to synthesize the literature, from coding themes, and describing themes to synthesizing themes (Bridges et al., 2020). Firstly, the researchers scrutinized the 53 enlightening studies included, sorting out the corresponding samples, research methods, analytical techniques, topics, and major findings. Secondly, the researchers classified the topics into six categories, academic self-concept, gender, achievement, motivation, performance, and self-efficacy. Finally, by illuminating the relevant research questions, the researchers made a comprehensive analysis of gender-moderated effects of academic self-concept on achievement, motivation, performance, and self-efficacy.

### Interpretation of included literature

**Figure 5:**
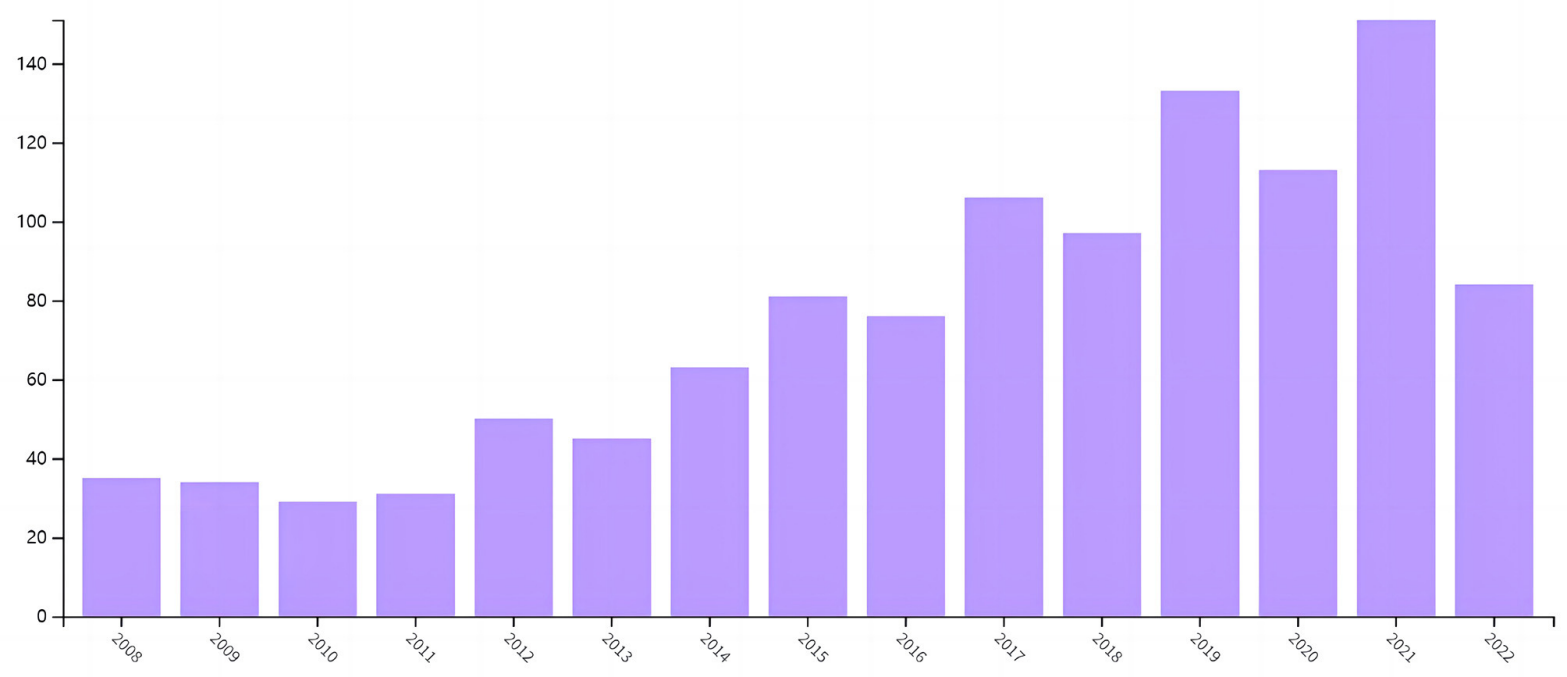
The number of publications on academic self-concept.

The included literature was listed alphabetically by the author's family name (see Appendix). All the enlightening literature included was published from 2008 to 2022. As shown by Figure 5, the academic researches on academic self-concept have been relatively stable before 2019, and the outbreak of the COVID-19 pandemic triggered the upsurge of studies on academic self-concept in 2021. This might be largely attributed to the constructive role of academic self-concept on educational constructs, such as achievement, performance, motivation, and self-efficacy, especially in adverse circumstances (Paechter et al., 2022).

The included literature could be interpreted from the perspective of samples, methods, and analytical techniques. As for samples, a majority of literature (*N* = 33) targeted at junior and senior middle school students. Only two studies explored the effects of academic self-concept among kindergarten students. Germany (*N* = 16) has topped the list for conducting researches on academic self-concept, followed by the United States (*N* = 4), Belgium (*N* = 3), and China (*N* = 3). Moreover, datasets involving multiple countries were employed in some literature, such as PISA and TIMSS. Questionnaires (*N* = 31), and scales (*N* = 18) were widely used in the researches. It was the structural equation model (*N* = 13) that explored gender-moderated effects of academic self-concept most effectively. Confirmatory factor analysis (*N* = 10) came second.

## Results

This section summarized the gender-moderated effects of academic self-concept on the following educational constructs, such as achievement, motivation, performance, and self-efficacy.

### RQ1: Can academic self-concept influence achievement?

Academic self-concept exerted a positive influence on individuals' achievement. Positive academic self-concept could boost achievement of both regular students and gifted students (Preckel et al., 2017). With higher academic self-concept, students' foreign language learning could be greatly facilitated, and academic achievement could also be enhanced (Zhang, 2022). As for college students, higher academic self-concept was conducive to an increase in classroom participation and achievement (Zhou et al., 2015). Driven by positive academic self-concept, students actively involved themselves in classroom activities, achieving more academic goals (Schnitzler et al., 2021). Moreover, teachers' higher expectations could also be converted into students' positive academic self-concept, contributing to satisfactory achievement (Szumski and Karwowski, 2019).

### Different findings

People took it for granted that the interrelation between academic self-concept and achievement should be linear, continuous, and stable. Nevertheless, it was not the case. Firstly, researches showed that there existed a non-linear rather than corresponding tendency between academic self-concept and achievement (Keller et al., 2021). This was because some under-achieving students tended to adopt self-protective strategies, pretending to hold a positive academic self-concept (Keller et al., 2021). Secondly, according to an investigation among Flemish adolescents, there was no such correlation between academic self-concept and achievement during a period ranging from grade 7 to grade 8 (Pinxten et al., 2013). Thirdly, the interrelation between academic self-concept and achievement was proven to change over time, varying from the skill-development model to the reciprocal effect model (Wu et al., 2021). Finally, cross-cultural modesty bias may make academic self-concept negatively associated with achievement (Min et al., 2016).

### RQ2: Can academic self-concept influence motivation?

Academic self-concept and motivation were interrelated with each other (Paechter et al., 2022). Students' high academic self-concept was generally accompanied by robust motivation while students' low academic self-concept could bring about declining motivation (Van de gaer et al., 2009). Students with robust motivation and positive academic self-concept tended to be willingly involved in academic activities (Burger and Naude, 2020). Freshmen academic self-concept was critical to subsequent motivation during university (Fryer, 2015). The gifted students with higher mathematical ability self-concept tended to hold higher intrinsic motivation (Bergold et al., 2020). Academic self-concept was proven to account for Japanese students' motivational deficits in learning English (Fryer et al., 2018).

### RQ3: Can academic self-concept influence performance?

Academic self-concept could boost students' performance. It has been confirmed that academic self-concept was conducive to promoting Spanish heritage learners' performance in reading, writing, and spelling (Beaudrie, 2018). Higher academic self-concept encouraged lower-achieving students to seek help from teachers and peers, which subsequently promoted students' achievement (Amemiya and Wang, 2017). In active learning classrooms, students with positive academic self-concept were more likely to participate in group discussions and achieve academic success (Cooper et al., 2018). By virtue of positive academic self-concept, Chinese Miao students' test anxiety was alleviated, and mathematical problem-solving performance was dramatically enhanced (Guo et al., 2020). Academic self-concept was demonstrated to explain course attendance and absence (Fryer et al., 2018) and make difference to students' course selection and dropout (Gorges, 2019).

### Inconsistent findings

Disagreement arose about whether academic self-concept could directly predict performance. Contrary to the mainstream studies, some researchers claimed that mathematics self-concept was incapable of directly predicting mathematics performance without mediation of mathematics self-efficacy and anxiety (Ferla et al., 2009). It was often the case that students suffering from test anxiety hold negative academic self-concept (von der Embse et al., 2018). Nevertheless, evidence showed that academic self-concept may not be capable of mediating the association between performance and test anxiety (Raymo et al., 2019).

### RQ4: Can academic self-concept influence self-efficacy?

Academic self-concept could exert a strong impact on self-efficacy but the reverse is not true (Arens et al., 2022). Because academic self-concept was oriented toward the past while self-efficacy was relevant to the future (Scherrer and Preckel, 2019). Take math as an example, although there was a positive association between previous self-concept and subsequent self-efficacy, no correlation was found between previous self-efficacy and subsequent self-concept (Arens et al., 2022). Students possessing higher academic self-concept in the active-learning environment were more likely to show a higher sense of self-efficacy and belonging (Aguillon et al., 2020). Academic self-concept was conducive to sustaining self-efficacy beliefs about maintaining interest in a specific domain (Fryer and Ainley, 2019).

### Controversies

It remained controversial whether the relationship between academic self-concept and self-efficacy was bidirectional or unidirectional. Some researchers claimed that academic self-concept and self-efficacy could mutually influence each other (Scherer, 2013). Nevertheless, it should be emphasized that self-efficacy merely exerted a temporary rather than permanent effect on academic self-concept (Ferla et al., 2009). Moreover, it was reported that enrollment in multiple developmental courses only exerted a negative influence on students' academic self-concept but made no difference to students' self-efficacy (Martin et al., 2017).

### RQ 5: Can gender differences moderate the effects of academic self-concept on achievement, motivation, performance, and self-efficacy?

To sum up, academic self-concept could exert positive influence on achievement, motivation, performance, and self-efficacy. Nevertheless, special attention should be paid to gender differences, since gender may play moderating roles in the effects of academic self-concept on the educational constructs mentioned above.

### Gender-moderated effects on achievement

Controversies have arisen as for whether gender differences could moderate the effect of academic self-concept on achievement. On one hand, some studies maintained that the correlation between academic self-concept and achievement was irrelevant to gender (Niepel et al., 2022), especially among youth with intellectual disabilities (Maïano et al., 2019). On the other hand, gender differences were recognized as for causality between academic self-concept and achievement (Pinxten et al., 2013). Compared with male counterparts, female students with a lower level of physics self-concept tended to be underachievers in physics (Hofer and Stern, 2016). Moreover, there were obvious gender discrepancies in math interest and academic self-concept since elementary school, which may bring about a large gap in math achievement (Ganley and Lubienski, 2016).

### Gender-moderated effects on motivation

Gender differences moderated the effects of academic self-concept on students' motivation. The school subjects in which students were motivated seemed to be consistent with gender stereotypes (Wirthwein et al., 2020). On one hand, boys tended to maintain higher motivation than girls, particularly in math and science (Dietrich and Lazarides, 2019; Espinoza and Taut, 2020). On the other hand, female students were more motivated in the verbal-related subjects, such as German and English (Wirthwein et al., 2020) and may excel in reading motivation (Muntoni and Retelsdorf, 2019). Moreover, girls were proven to be more motivated in learning than boys, which in turn may bring about better academic performance (Alivernini et al., 2020). Girls' higher motivation may be attributed to their characteristics. Compared with boys, girls were reported to care more about satisfying social acceptance and teachers' feedback (Brass et al., 2019).

### Gender-moderated effects on performance

Gender was a moderator on the effects of academic self-concept on students' performance. Gender identity made a difference to students' performance (Aguillon et al., 2020). Assuming STEM courses to be male-dominated exerted a negative influence on female students' academic self-concept in the physics and chemistry domain (Cooper et al., 2018), which in turn caused female students' passive participation (Sax et al., 2017), under-representation in STEM (Else-Quest et al., 2013) and Olympiads (Steegh et al., 2019). Male students participated more actively and frequently in active-learning classrooms (Aguillon et al., 2020). Gender differences also applied to gifted students, gifted boys outperformed gifted girls in all tasks, except verbal tasks (Gindi et al., 2019). Girls had no other choice but to spare more effort to study, striving for better performance (Van Houtte, 2017). Moreover, in terms of academic adjustment, male students were more adaptive to transition to higher education (Rosman et al., 2020).

### Gender-moderated effects on self-efficacy

There were inconsistent findings about whether gender differences exerted an influence on self-efficacy. Some researchers insisted that gender effects were found on mathematics self-efficacy but not science self-efficacy (Louis and Mistele, 2012; Chen and Usher, 2013). Other researchers maintained that there existed a deep-rooted gender stereotype of self-efficacy, especially in STEM courses (Aguillon et al., 2020). In other words, male students seemed to maintain higher mathematics self-concept and self-efficacy than female students (Bakan Kalaycioglu, 2017). Female students were more susceptible to gender identity and held lower self-efficacy in STEM courses (Aguillon et al., 2020). Some researches attributed this phenomenon to girls' academic preferences. Girls' math self-concept and self-efficacy were dwarfed by their outstanding verbal self-concept and self-efficacy (Marsh et al., 2019), which belittled girls' academic achievement.

## Discussion

This systematic review aimed at exploring the gender-moderated effects of academic self-concept on several significant and representative educational constructs, such as achievement, motivation, performance, and self-efficacy. The review was based on 53 included studies. Each research question received a thorough and satisfactory exploration. In a word, gender was an indispensable factor when it came to effects of academic self-concept on achievement, motivation, performance, and self-efficacy.

### The positive effects of academic self-concept

Academic self-concept played a crucial role in promoting students' educational outcomes. High levels of academic self-concept provided students the impetus to boost achievement, strengthen motivation, improve performance, and promote the cultivation of self-efficacy. The results presented in this research were compatible with the opinions proposed in the previous studies. High levels of academic self-concept enabled students to achieve goals smoothly and fulfill themselves (Berger et al., 2020). Positive academic self-concept may slow down the declining motivation in school careers (Scherrer and Preckel, 2019). Academic self-concept was conducive to strengthening the positive link between motivation and performance (Erentaite et al., 2022). Academic self-concept, such as computer thinking self-concept, was proven to be tightly associated with self-efficacy (Guggemos, 2021).

Nevertheless, it can't be taken for granted that linear, continuous, and stable relations remained constantly between academic self-concept and achievement. The self-protective strategies adopted by underachieving students may derange the linear relationship. The correlation between academic self-concept and achievement may fade away temporarily (Keller et al., 2021) or change over the school period (Pinxten et al., 2013). Academic self-concept and achievement turned out to be negatively correlated under modesty bias across cultures (Min et al., 2016).

### The gender-moderated effects of academic self-concept

An increasing body of studies has indicated that gender was an indispensable variable in educational researches (Espinoza and Taut, 2020). Compared with boys, girls were vulnerable to suffering from the threat of gender stereotypes (Aguillon et al., 2020). Boys and girls might behave differently in terms of academic self-concept, achievement, motivation, performance, and self-efficacy. Attitudes toward STEM courses differed from males to females, which accounted for gender disparities in classroom participation (Neill et al., 2019) and academic competitions (Steegh et al., 2019). It has also been confirmed that STEM-oriented self-concept could be negatively attributed to gender stereotypes (Ertl et al., 2017).

Gender differences could play moderating roles in the effects of academic self-concept on achievement, motivation, performance, and self-efficacy. The pervasive gender stereotypes or gender bias could impose an adverse influence on female students' academic self-concept, which may further influence their educational outcomes, undermining their confidence and impairing their self-efficacy (Ertl et al., 2017). Male students held higher mathematics self-concept, motivation, and self-efficacy (Arens et al., 2022) while female students bore themselves with higher verbal-related self-concept (Arens et al., 2018). Gender disparities in academic self-concept caused female students' passive participation (Sax et al., 2017) and under-representation in STEM (Else-Quest et al., 2013). Female students' lower STEM self-concept negatively affected their self-efficacy, which made them doubtful about their achievement, showing lower STEM success expectancy (Robnett and Thoman, 2017).

### Suggestions for educational instructors

In view of gender disparities in classroom participation, equitable teaching strategies should be adopted to maximize the benefits of active learning (Aguillon et al., 2020), making boys and girls equally involved in STEM courses. Taking gender differences into account, teachers should offer more help to female students, since girls are proven to be more dependent on teachers' instructional support (Espinoza and Taut, 2020). Early interventions should be implemented to boost female students' math interest, since gender discrepancies in math interest may lead to distinct math outcomes (Ganley and Lubienski, 2016). There is no denying that equitable teaching strategies may not be an absolute remedy. Teachers should implement flexible teaching strategies, in accordance with the distinct aptitude of students, to motivate different genders (Yu and Deng, 2022).

Moreover, it is advisable for instructors to reform academic training programs, by combining conventional teaching with hands-on activities. Practical experience is integral for students to foster self-identity, boost academic self-concept and self-efficacy. Encouraging students to participate in hands-on activities is conducive to building up female students' confidence, especially in disappointing and daunting STEM courses (Betz et al., 2021). Moreover, teachers should give more positive encouragement and feedback to female students, since gender bias may lead to an overestimation of male students' mathematics achievement and an underestimation of female students' mathematics achievement (McCoy et al., 2022). Improvement of academic self-concept may further enhance female students' learning motivation and interest.

### Educational policy implications

In view of the significance of academic self-concept in educational outcomes (Postigo et al., 2022), educational policies should be made and implemented to boost students' academic self-concept. Given the fact that ability stratification could make a difference to academic self-concept (Parker et al., 2021), within-school ability-streaming policy should be adopted flexibly and abstemiously to protect students from suffering from BFLPE (Liem et al., 2013). Moreover, in order to mitigate BFLPE, it was advisable to adopt more assessment tasks rather than tests or exams in both academically selective schools and comprehensive schools (Seaton et al., 2015). Active-learning pedagogy should be promoted in educational policies, encouraging students at all levels to participate in classroom activities (Aguillon et al., 2020).

Given the moderating role of gender in academic self-concept, it is essential to incorporate gender into educational policies (Furlin, 2021). Undoubtedly, gender stereotypes may hinder females from making excellent achievement in educational systems (Nakray, 2018). Therefore, it was incumbent on policymakers to make and carry out policies to ensure educational equality across genders. Educational policies should be made to promote inclusive education, paying enough attention to gender discrepancies in academic self-concept and minimizing gender stereotypes in educational settings (Alexiadou and Rambla, 2022). As for curriculum policy, courses should integrate learning orientation, and goal orientation with activity orientation, imperceptibly enhancing female students' STEM self-concept (Mynott, 2018). In addition, textbooks could be compiled with case studies of outstanding female representatives in STEM field (Espinoza and Taut, 2020).

## Conclusion

### Major findings

This study conducted a systematic review of the gender-moderated effects of academic self-concept on achievement, motivation, performance, and self-efficacy. In line with previous studies, this study firmly supported that academic self-concept exerted a positive influence on educational outcomes. Specifically speaking, academic self-concept could improve achievement, enhance motivation, ameliorate performance, and boost self-efficacy. Moreover, the interrelation between academic self-concept and achievement may not be linear, continuous, and stable. Additionally, it should also be highlighted that gender played moderating roles in the link between academic self-concept and achievement, motivation, performance, and self-efficacy. Gender stereotypes exerted a negative influence on female students' math-related self-concept and self-efficacy, causing female students to be underachievers in STEM courses and careers. Although female students were less motivated in math-related discipline, they spared more effort to cope with social comparison.

### Limitations

Frankly, several limitations remain unaddressed in this study. Firstly, due to the limited library resources, the only accessible database to this study is the authoritative Web of Science (core collection), causing that the literature retrieved may not be totally comprehensive. Secondly, in terms of scope field, this study exclusively narrows down the scope to education and educational research. Moreover, besides achievement, motivation, performance, and self-efficacy, there are many other educational constructs needed to be considered, such as academic buoyance, school burnout, attitude, interest, engagement, and attribution etc. Thirdly, taking the language mastery into consideration, the literature not written in English is excluded.

### Implications for future studies

Future studies should pay attention to promote educational equality in terms of gender differences. Measures should be taken to involve female students in class discussion and interaction. Equitable teaching strategies and academic training programs should be adopted to enhance female students' STEM self-concept. Moreover, teachers should design different courses based on different gender characteristics (Yu, 2021). Only gender differences are taken into consideration could education reforms achieve the goal of gender equality.

Future studies should attach importance to special groups, such as immigrant students, children of migrant workers, minority students, and students with special educational needs. With the development of globalization and urbanization, there is an increasing tendency of immigrant children and migrant children. However, immigrant students were vulnerable to anxiety and depression at school (Alivernini et al., 2020). Moreover, numerous studies targeted at regular students, overlooking the particularities of minority students and disabled students. The interrelations between academic self-concept and educational constructs among disabled students may be more complex than that among regular students (Maïano et al., 2019).

Future studies should explore how to promote students' academic self-concept in online learning. The rampant pandemic of COVID-19 compelled teachers and students to accept online learning as the main way of delivering lectures and acquiring knowledge (Yu et al., 2022). It was proven that a positive academic self-concept enabled students to meet the challenges imposed by COVID-19 (Paechter et al., 2022). However, online learning is undoubtedly distinct from traditional classroom learning. Since it has not reached a consistent conclusion as for the role of gender on online learning outcomes (Yu, 2021), gender-moderated effects of academic self-concept in an online learning context remain to be explored.

## Data availability statement

The original contributions presented in the study are included in the article/Supplementary material, further inquiries can be directed to the corresponding author.

## Author contributions

LW: methodology, investigation, editing, and writing—original draft. ZY: conceptualization and funding acquisition. Both authors listed have made a substantial, direct, and intellectual contribution to the work and approved it for publication.

## References

[B1] Aguillon S. M. Siegmund G.-F. Petipas R. H. Drake A. G. Cotner S. Ballen C. J. (2020). Gender differences in student participation in an active-learning classroom. CBE—Life Sci. Educ. 19, ar12. 10.1187/cbe.19-03-0048 32453677 PMC8697656

[B2] Alexiadou N. Rambla X. (2022). Education policy governance and the power of ideas in constructing the new European Education Area. Eur. J. Educ. Res. 1–18. 10.1177/14749041221121388

[B3] Alivernini F. Cavicchiolo E. Manganelli S. Chirico A. Lucidi F. (2020). Students' psychological wellbeing and its multilevel relationship with immigrant background, gender, socioeconomic status, achievement, and class size. Sch. Eff. Sch. Improv. 31, 172–191. 10.1080/09243453.2019.1642214

[B4] Amemiya J. Wang M.-T. (2017). Transactional relations between motivational beliefs and help seeking from teachers and peers across adolescence. J. Youth Adolesc. 46, 1743–1757. 10.1007/s10964-016-0623-y 27942932

[B5] Arens A. K. Becker M. Möller J. (2018). The internal/external frame of reference (I/E) model: Extension to five school subjects and invariance across German secondary school ability tracks. Learn. Individ. Differ. 67, 143–155. 10.1016/j.lindif.2018.07.005

[B6] Arens A. K. Frenzel A. C. Goetz T. (2022). Self-concept and self-efficacy in math: longitudinal interrelations and reciprocal linkages with achievement. The Journal of Experimental Education 90, 615–633. 10.1080/00220973.2020.1786347

[B7] Arens A. K. Jansen M. (2016). Self-concepts in reading, writing, listening, and speaking: a multidimensional and hierarchical structure and its generalizability across native and foreign languages. J. Educ. Psychol. 108, 646–664. 10.1037/edu0000081

[B8] Arens A. K. Möller J. Watermann R. (2016). Extending the internal/external frame of reference model to social studies: self-concept and achievement in history and politics. Learn. Individ. Differ. 51, 91–99. 10.1016/j.lindif.2016.08.044

[B9] Arens A. K. Preckel F. (2018). Testing the internal/external frame of reference model with elementary school children: extension to physical ability and intrinsic value. Contemp. Educ. Psychol. 54, 199–211. 10.1016/j.cedpsych.2018.06.003

[B10] Bakan Kalaycioglu D. (2017). The big fish-little pond effect on affective factors based on PISA 2012 mathematics achievement. Egitimde ve Psikolojide Ölçme ve Degerlendirme Dergisi 8, 1–1. 10.21031/epod.297686

[B11] Beaudrie S. M. (2018). On the relationship between self-concept and literacy development in the Spanish heritage language context. Read. Writ. Q. 34, 147–159. 10.1080/10573569.2017.1370623

[B12] Belfi B. Goos M. De Fraine B. Van Damme J. (2012). The effect of class composition by gender and ability on secondary school students' school wellbeing and academic self-concept: a literature review. Educ. Res. Rev. 7, 62–74. 10.1016/j.edurev.2011.09.002

[B13] Berger N. Mackenzie E. Holmes K. (2020). Positive attitudes towards mathematics and science are mutually beneficial for student achievement: a latent profile analysis of TIMSS 2015. Aust. Educ. Res. 47, 409–444. 10.1007/s13384-020-00379-8

[B14] Bergold S. Wirthwein L. Steinmayr R. (2020). Similarities and differences between intellectually gifted and average-ability students in school performance, motivation, and subjective wellbeing. Gifted Child Q. 64, 285–303. 10.1177/0016986220932533

[B15] Betz A. R. King B. Grauer B. Montelone B. Wiley Z. Thurston L. (2021). Improving academic self-concept and STEM identity through a research immersion: pathways to STEM summer program. Front. Educat. 6, 674817. 10.3389/feduc.2021.674817

[B16] Bieg M. Goetz T. Wolter I. Hall N. C. (2015). Gender stereotype endorsement differentially predicts girls' and boys' trait-state discrepancy in math anxiety. Front. Psychol. 6, 01404. 10.3389/fpsyg.2015.01404 26441778 PMC4585180

[B17] Booth A. (2006). “Brimful of STARLITE”: toward standards for reporting literature searches. J. Med. Library Assoc. 94, 421–e205. 17082834 PMC1629442

[B18] Brass N. McKellar S. E. North E. A. Ryan A. M. (2019). Early adolescents' adjustment at school: a fresh look at grade and gender differences. J. Early Adolesc. 39, 689–716. 10.1177/0272431618791291

[B19] Bridges J. Collins P. Flatley M. Hope J. Young A. (2020). Older people's experiences in acute care settings: systematic review and synthesis of qualitative studies. Int. J. Nurs. Stud. 102, 103469. 10.1016/j.ijnurstu.2019.103469 31862528

[B20] Brisson B. M. Dicke A.-L. Gaspard H. Häfner I. Flunger B. Nagengast B. . (2017). Short intervention, sustained effects: promoting students' math competence beliefs, effort, and achievement. Am. Educ. Res. J. 54, 1048–1078. 10.3102/0002831217716084

[B21] Burger A. Naudé L. (2019). Predictors of academic success in the entry and integration stages of students' academic careers. Social Psychol. Educ. 22, 743–755. 10.1007/s11218-019-09497-3

[B22] Burger A. Naude L. (2020). In their own words—Students' perceptions and experiences of academic success in higher education. Educ. Stud. 46, 624–639. 10.1080/03055698.2019.1626699

[B23] Cambria J. Brandt H. Nagengast B. Trautwein U. (2017). Frame of reference effects on values in mathematics: evidence from german secondary school students. ZDM. 49, 435–447. 10.1007/s11858-017-0841-0

[B24] Chen J. A. Usher E. L. (2013). Profiles of the sources of science self-efficacy. Learn. Individ. Differ. 24, 11–21. 10.1016/j.lindif.2012.11.002

[B25] Chen S.-K. Hwang F.-M. Yeh Y.-C. Lin S. S. J. (2012). Cognitive ability, academic achievement and academic self-concept: extending the internal/external frame of reference model: extending the internal/external frame of reference model. Br. J. Educ. Psychol. 82, 308–326. 10.1111/j.2044-8279.2011.02027.x 22583093

[B26] Cheung D. (2018). The key factors affecting students' individual interest in school science lessons. Int. J. Sci. Educ. 40, 1–23. 10.1080/09500693.2017.1362711

[B27] Colmar S. Liem G. A. D. Connor J. Martin A. J. (2019). Exploring the relationships between academic buoyancy, academic self-concept, and academic performance: a study of mathematics and reading among primary school students. Educ. Psychol. 39, 1068–1089. 10.1080/01443410.2019.1617409

[B28] Cooper K. M. Krieg A. Brownell S. E. (2018). Who perceives they are smarter? Exploring the influence of student characteristics on student academic self-concept in physiology. Adv. Physiol. Educ. 42, 200–208. 10.1152/advan.00085.2017 29616569

[B29] Dietrich J. Lazarides R. (2019). Gendered development of motivational belief patterns in mathematics across a school year and career plans in math-related fields. Front. Psychol. 10, 1472. 10.3389/fpsyg.2019.01472 31316432 PMC6610766

[B30] Dijkstra P. Kuyper H. van der Werf G. Buunk A. P. van der Zee Y. G. (2008). Social comparison in the classroom: a review. Rev. Educ. Res. 78, 828–879. 10.3102/0034654308321210

[B31] Else-Quest N. M. Mineo C. C. Higgins A. (2013). Math and science attitudes and achievement at the intersection of gender and ethnicity. Psychol. Women Q. 37, 293–309. 10.1177/0361684313480694

[B32] Emmerichs L. Welter V. D. E. Schlüter K. (2021). University teacher students' learning in times of COVID-19. Educ. Sci. 11, 776. 10.3390/educsci11120776

[B33] Erentaite R. Vosylis R. Sevalneva D. Melnikė E. RaiŽienė S. Daukantaite D. (2022). Profiles of achievement motivation and performance in middle school: links to student background and perceived classroom climate. Front. Psychol. 13, 820247. 10.3389/fpsyg.2022.820247 35707671 PMC9191575

[B34] Ertl B. Luttenberger S. Paechter M. (2017). The impact of gender stereotypes on the self-concept of female students in STEM subjects with an under-representation of females. Front. Psychol. 8, 703. 10.3389/fpsyg.2017.00703 28567022 PMC5434750

[B35] Espinoza A. M. Taut S. (2020). Gender and psychological variables as key factors in mathematics learning: a study of seventh graders in Chile. Int. J. Educ. Res. 103, 101611. 10.1016/j.ijer.2020.101611

[B36] Fadda D. Pellegrini M. Vivanet G. Zandonella Callegher C. (2022). Effects of digital games on student motivation in mathematics: a meta-analysis in K-12. J. Comp. Assisted Learn. 38, 304–325. 10.1111/jcal.12618

[B37] Ferla J. Valcke M. Cai Y. (2009). Academic self-efficacy and academic self-concept: reconsidering structural relationships. Learn. Individ. Differ. 19, 499–505. 10.1016/j.lindif.2009.05.004

[B38] Friedrich A. Flunger B. Nagengast B. Jonkmann K. Trautwein U. (2015). Pygmalion effects in the classroom: teacher expectancy effects on students' math achievement. Contemp. Educ. Psychol. 41, 1–12. 10.1016/j.cedpsych.2014.10.006

[B39] Fryer L. K. (2015). Predicting self-concept, interest and achievement for first-year students: the seeds of lifelong learning. Learn. Individ. Differ. 38, 107–114. 10.1016/j.lindif.2015.01.007

[B40] Fryer L. K. Ainley M. (2019). Supporting interest in a study domain: a longitudinal test of the interplay between interest, utility-value, and competence beliefs. Learning and Instruction 60, 252–262. 10.1016/j.learninstruc.2017.11.002

[B41] Fryer L. K. Ginns P. Howarth M. Anderson C. Ozono S. (2018). Individual differences and course attendance: why do students skip class? Educational Psychology 38, 470–486. 10.1080/01443410.2017.1403567

[B42] Furlin N. (2021). From gender to ‘gender ideology' in the field of educational policies: theoretical, historical and political notes. Revista Praxis Educacional. 17, 44. 10.22481/praxisedu.v17i44.7042

[B43] Ganley C. M. Lubienski S. T. (2016). Mathematics confidence, interest, and performance: examining gender patterns and reciprocal relations. Learn. Individ. Differ. 47, 182–193. 10.1016/j.lindif.2016.01.002

[B44] Gindi S. Kohan-Mass J. Pilpel A. (2019). Gender differences in competition among gifted students: the role of single-sex vs. co-ed classrooms. Roeper Rev. 41, 199–211. 10.1080/02783193.2019.1622163

[B45] Gorges J. (2019). Motivational beliefs specific to business studies subfields: interrelations, antecedents, and change in the introductory study phase. Int. J.Educ. Psychol. 8, 109. 10.17583/ijep.2019.3780

[B46] Gorges J. Neumann P. Wild E. Stranghöner D. Lütje-Klose B. (2018). Reciprocal effects between self-concept of ability and performance: a longitudinal study of children with learning disabilities in inclusive vs. exclusive elementary education. Learn. Individ. Differ. 61, 11–20. 10.1016/j.lindif.2017.11.005

[B47] Guggemos J. (2021). On the predictors of computational thinking and its growth at the high-school level. Comput. Educ. 161, 104060. 10.1016/j.compedu.2020.104060

[B48] Guo M. Leung F. K. S. Hu X. (2020). Affective determinants of mathematical problem posing: the case of Chinese Miao students. Educ. Stud. Mathemat. 105, 367–387. 10.1007/s10649-020-09972-1

[B49] Hofer S. I. Stern E. (2016). Underachievement in physics: when intelligent girls fail. Learn. Individ. Differ. 51, 119–131. 10.1016/j.lindif.2016.08.006

[B50] Jansen M. Lüdtke O. Robitzsch A. (2020). Disentangling different sources of stability and change in students' academic self-concepts: an integrative data analysis using the STARTS model. J. Educ. Psychol. 112, 1614–1631. 10.1037/edu0000448

[B51] Jónsdóttir H. H. Blöndal K. S. (2022). The choice of track matters: academic self-concept and sense of purpose in vocational and academic tracks. Scand. J. Educational Res. 1–16. 10.1080/00313831.2022.2042843

[B52] Kaur T. McLoughlin E. Grimes P. (2022). Mathematics and science across the transition from primary to secondary school: a systematic literature review. Int. J. STEM Educ. 9, 13. 10.1186/s40594-022-00328-0

[B53] Kavanagh L. (2020). Academic self-concept formation: Testing the internal/external frame of reference model, big-fish-little-pond model, and an integrated model at the end of primary school. Eur. J.Psychol. Educat. 35, 93–109. 10.1007/s10212-019-00416-w

[B54] Keller L. Preckel F. Brunner M. (2021). Nonlinear relations between achievement and academic self-concepts in elementary and secondary school: an integrative data analysis across 13 countries. J. Educ. Psychol. 113, 585–604. 10.1037/edu0000533

[B55] Koivuhovi S. Vainikainen M.-P. Kalalahti M. Niemivirta M. (2019). Changes in children's agency beliefs and control expectancy in classes with and without a special emphasis in finland from grade four to grade six. Scand. J. Educ. Res. 63, 427–442. 10.1080/00313831.2017.1402364

[B56] Liem G. A. D. Marsh H. W. Martin A. J. McInerney D. M. Yeung A. S. (2013). The big-fish-little-pond effect and a national policy of within-school ability streaming: Alternative frames of reference. Am. Educ. Res. J. 50, 326–370. 10.3102/0002831212464511

[B57] Lohbeck A. Möller J. (2017). Social and dimensional comparison effects on math and reading self-concepts of elementary school children. Learn. Individ. Differ. 54, 73–81. 10.1016/j.lindif.2017.01.013

[B58] Louis R. A. Mistele J. M. (2012). The differences in scores and self-efficacy by student gender in mathematics and science. Int. J. Sci. Math. 10, 1163–1190. 10.1007/s10763-011-9325-9

[B59] Maïano C. Coutu S. Morin A. J. S. Tracey D. Lepage G. Moullec G. (2019). Self-concept research with school-aged youth with intellectual disabilities: a systematic review. J. Appl. Res. Intellect. Disabil. 32, 238–255. 10.1111/jar.12543 30515961

[B60] Marsh H. W. Kuyper H. Seaton M. Parker P. D. Morin A. J. S. Möller J. . (2014). Dimensional comparison theory: an extension of the internal/external frame of reference effect on academic self-concept formation. Contemp. Educ. Psychol. 39, 326–341. 10.1016/j.cedpsych.2014.08.003

[B61] Marsh H. W. Martin A. J. (2011). Academic self-concept and academic achievement: Relations and causal ordering: academic self-concept. Br. J. Educational Psychol. 81, 59–77. 10.1348/000709910X503501 21391964

[B62] Marsh H. W. Van Zanden B. Parker P. D. Guo J. Conigrave J. Seaton M. (2019). Young women face disadvantage to enrollment in university STEM coursework regardless of prior achievement and attitudes. Am. Educ. Res. J. 56, 1629–1680. 10.3102/0002831218824111

[B63] Martin K. Goldwasser M. Harris E. (2017). Developmental education's impact on students' academic self-concept and self-efficacy. J. College Student Retent. 18, 401–414. 10.1177/152102511560485028533360

[B64] McCoy S. Byrne D. O'Connor P. (2022). Gender stereotyping in mothers' and teachers' perceptions of boys' and girls' mathematics performance in Ireland. Oxford Rev. Educ. 48, 341–363. 10.1080/03054985.2021.1987208

[B65] Min I. Cortina K. S. Miller K. F. (2016). Modesty bias and the attitude-achievement paradox across nations: a reanalysis of TIMSS. Learn. Individ. Differ. 51, 359–366. 10.1016/j.lindif.2016.09.008

[B66] Muntoni F. Retelsdorf J. (2019). At their children's expense: how parents' gender stereotypes affect their children's reading outcomes. Learn. Instruct. 60, 95–103. 10.1016/j.learninstruc.2018.12.002

[B67] Mynott G. J. (2018). The academic self-concept of business and management students: a review of the literature. Int. J. Manag. Educ. 16, 515–523. 10.1016/j.ijme.2018.10.003

[B68] Nakray K. (2018). Gender and education policy in India: twists, turns and trims of transnational policy transfers. Int. Sociol. 33, 27–44. 10.1177/0268580917745769

[B69] Neill C. Cotner S. Driessen M. J. Ballen C. (2019). Structured learning environments are required to promote equitable participation. Chem. Educ. Res. Pract. 20, 197–203. 10.1039/C8RP00169C

[B70] Niepel C. Marsh H. W. Guo J. Pekrun R. Möller J. (2022). Revealing dynamic relations between mathematics self-concept and perceived achievement from lesson to lesson: An experience-sampling study. J. Educ. Psychol. 114, 1380–1393. 10.1037/edu0000716

[B71] Paechter M. Phan-Lesti H. Ertl B. Macher D. Malkoc S. Papousek I. (2022). Learning in adverse circumstances: impaired by learning with anxiety, maladaptive cognitions, and emotions, but supported by self-concept and motivation. Front. Psychol. 13, 850578. 10.3389/fpsyg.2022.850578 35496215 PMC9046842

[B72] Parker P. Dicke T. Guo J. Basarkod G. Marsh H. (2021). Ability stratification predicts the size of the big-fish-little-pond effect. Educ. Res. 50, 334–344. 10.3102/0013189X20986176

[B73] Pinxten M. De Fraine B. Van Damme J. D'Haenens E. (2013). Student achievement and academic self-concept among secondary students in Flanders: gender and changes over time. Irish Educ. Stud. 32, 157–178. 10.1080/03323315.2012.749058

[B74] Postigo Á. Fernández-Alonso R. Fonseca-Pedrero E. González-Nuevo C. Muñiz J. (2022). Academic self-concept dramatically declines in secondary school: personal and contextual determinants. Int. J. Environ. Res. Public Health 19, 3010. 10.3390/ijerph19053010 35270703 PMC8910088

[B75] Preckel F. Goetz T. Pekrun R. Kleine M. (2008). Gender differences in gifted and average-ability students: comparing girls' and boys' achievement, self-concept, interest, and motivation in mathematics. Gifted Child Q. 52, 146–159. 10.1177/0016986208315834

[B76] Preckel F. Schmidt I. Stumpf E. Motschenbacher M. Vogl K. Schneider W. (2017). A test of the reciprocal-effects model of academic achievement and academic self-concept in regular classes and special classes for the gifted. Gifted Child Q. 61, 103–116. 10.1177/0016986216687824

[B77] PRISMA-P Group Moher D. Shamseer L. Clarke M. Ghersi D. Liberati A. . (2015). Preferred reporting items for systematic review and meta-analysis protocols (PRISMA-P) 2015 statement. Syst. Rev. 4, 1. 10.1186/2046-4053-4-1 25554246 PMC4320440

[B78] Raymo L. A. Somers C. L. Partridge R. T. (2019). Adolescent test anxiety: an examination of intraindividual and contextual predictors. School Ment. Health 11, 562–577. 10.1007/s12310-018-09302-0

[B79] Robnett R. D. Thoman S. E. (2017). STEM success expectancies and achievement among women in STEM majors. J. Appl. Dev. Psychol. 52, 91–100. 10.1016/j.appdev.2017.07.003

[B80] Rosman T. Mayer A.-K. Leichner N. Krampen G. (2020). Putting big fish into a bigger pond: self-concept changes in psychology undergraduate entrants. J. Further Higher Educ. 44, 14–28. 10.1080/0309877X.2018.1493095

[B81] Safavian N. (2019). What makes them persist? Expectancy-value beliefs and the math participation, performance, and preparedness of hispanic youth. AERA Open. 5, 233285841986934. 10.1177/2332858419869342

[B82] Savolainen P. A. Timmermans A. C. Savolainen H. K. (2018). Part-time special education predicts students' reading self-concept development. Learn. Individ. Differ. 68, 85–95. 10.1016/j.lindif.2018.10.005

[B83] Sax L. J. Lehman K. J. Jacobs J. A. Kanny M. A. Lim G. Monje-Paulson L. . (2017). Anatomy of an enduring gender gap: the evolution of women's participation in computer science. J. Higher Educ. 88, 258–293. 10.1080/00221546.2016.1257306

[B84] Scherer R. (2013). Further evidence on the structural relationship between academic self-concept and self-efficacy: on the effects of domain specificity. Learn. Individ. Differ. 28, 9–19. 10.1016/j.lindif.2013.09.008

[B85] Scherrer V. Preckel F. (2019). Development of motivational variables and self-esteem during the school career: a meta-analysis of longitudinal studies. Rev. Educ. Res. 89, 211–258. 10.3102/0034654318819127

[B86] Schnitzler K. Holzberger D. Seidel T. (2021). All better than being disengaged: student engagement patterns and their relations to academic self-concept and achievement. Eur. J. Psychol. Educ. 36, 627–652. 10.1007/s10212-020-00500-6

[B87] Seaton M. Marsh H. W. Parker P. D. Craven R. G. Yeung A. S. (2015). The reciprocal effects model revisited: extending its reach to gifted students attending academically selective schools. Gifted Child Q. 59, 143–156. 10.1177/0016986215583870

[B88] Shavelson R. J. Hubner J. J. Stanton G. C. (1976). Self-concept: validation of construct interpretations. Rev. Educ. Res. 46, 407–441. 10.3102/00346543046003407

[B89] Steegh A. M. Höffler T. N. Keller M. M. Parchmann I. (2019). Gender differences in mathematics and science competitions: a systematic review. J. Res. Sci. Teach. 56, 1431–1460. 10.1002/tea.21580

[B90] Steinmayr R. Weidinger A. F. Wigfield A. (2018). Does students' grit predict their school achievement above and beyond their personality, motivation, and engagement? Contemp. Educ. Psychol. 53, 106–122. 10.1016/j.cedpsych.2018.02.004

[B91] Szumski G. Karwowski M. (2019). Exploring the pygmalion effect: the role of teacher expectations, academic self-concept, and class context in students' math achievement. Contemp. Educ. Psychol. 59, 101787. 10.1016/j.cedpsych.2019.101787

[B92] Tomás J. M. Gutiérrez M. Georgieva S. Hernández M. (2020). The effects of self-efficacy, hope, and engagement on the academic achievement of secondary education in the Dominican Republic. Psychol. Sch. 57, 191–203. 10.1002/pits.22321

[B93] Valls M. (2022). Gender differences in social comparison processes and self-concept among students. Front. Educ. 6, 815619. 10.3389/feduc.2021.815619

[B94] Van de gaer E. De Fraine B. Pustjens H. Van Damme J. De Munter A. Onghena P. (2009). School effects on the development of motivation toward learning tasks and the development of academic self-concept in secondary education: a multivariate latent growth curve approach. Sch. Eff. Sch. Improv. 20, 235–253. 10.1080/09243450902883920

[B95] Van Houtte M. (2017). Gender differences in context: the impact of track position on study involvement in flemish secondary education. Sociol. Educ. 90, 275–295. 10.1177/0038040717731604

[B96] von der Embse N. Jester D. Roy D. Post J. (2018). Test anxiety effects, predictors, and correlates: a 30-year meta-analytic review. J. Affect. Disord. 227, 483–493. 10.1016/j.jad.2017.11.048 29156362

[B97] Vu T. Magis-Weinberg L. Jansen B. R. J. van Atteveldt N. Janssen T. W. P. Lee N. C. . (2022). Motivation-achievement cycles in learning: a literature review and research agenda. Educ. Psychol. Rev. 34, 39–71. 10.1007/s10648-021-09616-7

[B98] Wan S. Lauermann F. Bailey D. H. Eccles J. S. (2021). When do students begin to think that one has to be either a “math person” or a “language person”? A meta-analytic review. Psychol. Bull. 147, 867–889. 10.1037/bul0000340

[B99] Wigfield A. Eccles J. S. (2000). Expectancy–value theory of achievement motivation. Contemp. Educ. Psychol. 25, 68–81. 10.1006/ceps.1999.1015 10620382

[B100] Wigfield A. Eccles J. S. Möller J. (2020). How dimensional comparisons help to understand linkages between expectancies, values, performance, and choice. Educ. Psychol. Rev. 32, 657–680. 10.1007/s10648-020-09524-2

[B101] Wirthwein L. Sparfeldt J. R. Heyder A. Buch S. R. Rost D. H. Steinmayr R. (2020). Sex differences in achievement goals: do school subjects matter? Eur. J. Psychol. Educ. 35, 403–427. 10.1007/s10212-019-00427-7

[B102] Wolff F. (2021). How classmates' gender stereotypes affect students' math self-concepts: a multilevel analysis. Front. Psychol. 12, 599199. 10.3389/fpsyg.2021.599199 34054632 PMC8149781

[B103] Wolff F. Möller J. (2022). An individual participant data meta-analysis of the joint effects of social, dimensional, and temporal comparisons on students' academic self-concepts. Educ. Psychol. Rev. 10.1007/s10648-022-09686-1

[B104] Wu H. Guo Y. Yang Y. Zhao L. Guo C. (2021). A meta-analysis of the longitudinal relationship between academic self-concept and academic achievement. Educ. Psychol. Rev. 33, 1749–1778. 10.1007/s10648-021-09600-1

[B105] Yu Z. (2021). The effects of gender, educational level, and personality on online learning outcomes during the COVID-19 pandemic. Int. J. Educ. Technol. 18, 14. 10.1186/s41239-021-00252-3 34778520 PMC8016506

[B106] Yu Z. Deng X. (2022). A meta-analysis of gender differences in e-learners' self-efficacy, satisfaction, motivation, attitude, and performance across the world. Front. Psychol. 13, 897327. 10.3389/fpsyg.2022.897327 35664150 PMC9159470

[B107] Yu Z. Xu W. Yu L. (2022). Constructing an online sustainable educational model in COVID-19 pandemic environments. Sustainability. 14, 3598. 10.3390/su1406359834962950

[B108] Zhang J. (2022). The impact of positive mood and future outlook on english as a foreign language students' academic self-concept. Front. Psychol. 13, 846422. 10.3389/fpsyg.2022.846422 35222219 PMC8873524

[B109] Zhou Y.-X. Ou C.-Q. Zhao Z.-T. Wan C.-S. Guo C. Li L. . (2015). The impact of self-concept and college involvement on the first-year success of medical students in China. Adv. Health Sci. Educ. 20, 163–179. 10.1007/s10459-014-9515-7 24906461

