# Supplementary material for: Gender-moderated effects of academic self-concept on achievement, motivation, performance, and self-efficacy: A systematic review
Source: Front Psychol. 2023 Mar 28;14:1136141. doi: 10.3389/fpsyg.2023.1136141 (PMC10086360; doi:10.3389/fpsyg.2023.1136141)
Supplement: Supplementary file 1 [file Table_1.docx]

**Appendix**

*Table Enlightening Studies included*

| **N** | **Author** | **Samples** | **Methods** | **Analytical** **techniques** | **Topics** | **Findings** |
| --- | --- | --- | --- | --- | --- | --- |
| 1 | Aguillon et al. (2020) | 509 American university students | survey, scale | confirmatory factor analysis | academic self-concept, self-efficacy | There existed a deep-rooted gender stereotype of self-efficacy, especially in STEM courses. |
| 2 | Arens et al. (2022) | 3209 German high school students | scale | structural equation modeling, Mplus 8.2 | academic self-concept, self-efficacy | As for math, the previous self-concept was positively associated with subsequent self-efficacy, whereas previous self-efficacy was not relevant to the subsequent self-concept. |
| 3 | Arens et al (2018) | 514 German elementary school students | questionnaire, scale | confirmatory factor analysis | academic self-concept, achievement | Academic self-concept was significantly interrelated with achievement within domains and across domains. |
| 4 | Bakan Kalaycioglu (2017) | 771 Turkish high school students | questionnaire | factor analysis; multiple indicator multiple cause model | achievement, academic self-concept | The increase of school’s mathematics achievement leads to a decrease of students’ mathematics self-concept. |
| 5 | Beaudrie (2018) | 281 Spanish primary school students | questionnaire | cluster analysis | academic self-concept, performance | Academic self-concept is conducive to improve students’ literacy performance. |
| 6 | Berger (2020) | 10051 Australian high school students | questionnaire | latent profile analysis | academic self-concept, achievement, performance | Positive self-concept leads to higher achievement and subjects preference towards math and science. |
| 7 | Burger et al. (2019) | 164 South African high school students | survey | standard multiple regression analyses | academic self-concept, performance | Academic self-concept and performance play a crucial in boosting students’ academic success. |
| 8 | Castejon (2016) | 1400 Spanish high school students | questionnaire | multiple regression analysis, Chi-square test | academic self-concept, achievement | Compared with underachieving and normal achieving students, overachieving students have higher academic self-concept. |
| 9 | Cambria et al. (2017) | 2079 German secondary school students | test, questionnaire | multilevel structural equation model | academic self-concept, achievement | Boys and girls underwent big-fish-little-pond effect in the same way. |
| 10 | Chen et al. (2015) | 407 Chinese vocational college students | questionnaire | structural equation modeling | academic self-concept, achievement | Learning strategies play a mediational role between academic self-concept and achievement. |
| 11 | Cheng et al. (2014) | 7334 Chinese secondary school students | questionnaire | confirmatory factor analysis | academic self-concept, motivation | Students with higher motivation suffer from stronger BFLPE. |
| 12 | Colmar et al. (2019) | 191 Australian primary school students | questionnaire, scale | structural equation model | academic self-concept, performance | Academic performance is associated with academic buoyancy via academic-self-concept. |
| 13 | Cooper (2018) | 244 American university students | survey | linear regression, logistic regression | academic self-concept, performance, achievement | Academic self-concept has an influence on students’ participation in group discussion and achievement in active learning. |
| 14 | de Gaer et al. (2009) | 2687 Belgian secondary school students | questionnaire, test, scale | multivariate multilevel latent growth curve model | Academic self-concept, motivation | There is a correlation between the development of motivation and academic self-concept. |
| 15 | Dicke et al. (2018) | 14,985 U.S. kindergartners | questionnaire | Chi-square test, | academic self-concept, achievement | School-average achievement has a negative effect on individual self-concept and no positive effect on individual achievement. |
| 16 | Dietrich et al. (2019) | 751 German secondary school students | scale | latent-transition analysis | motivation | Boys tended to maintain higher motivation than girls particularly in math and science. |
| 17 | Emmerichs, L., et al. (2021) | 395 German teacher students | survey | multivariate analysis of variance, correlational analyses | academic self-concept | The COVID-19 pandemic exerted a negative effect on students’ academic self-concept. |
| 18 | Erentaitė et al., 2022 | 1439 Lithuanian secondary school students | test, scale, questionnaire | latent profile analysis | academic self-concept, motivation, performance | Academic self-concept was conducive to strengthen the positive link between motivation and performance. |
| 19 | Ertl et al. (2017) | 296 German university students | survey | latent regression model | academic self-concept, performance | STEM-oriented self-concept could be negatively attributed to gender stereotypes. |
| 20 | Espinoza et al. (2020) | 1380 Chilean secondary school students | test, questionnaire | multivariate analysis, multi-level regression analyses | academic self-concept, motivation, achievement | There exist gender differences in mathematics learning, namely, boys surpass girls in mathematics academic self-concept, achievement, and motivation. |
| 21 | Ferla et al. (2009) | 8796 Belgian secondary school students | survey | confirmatory factor analysis | academic self-concept, self-efficacy, achievement | Students’ academic self-concept has an influence on their self-efficacy beliefs and academic self-efficacy could predict academic achievement. |
| 22 | Ferla et al. (2010). | 512 Belgian freshmen | questionnaire | confirmatory factor analysis | academic self-concept, self-efficacy, performance, motivation | Both academic self-concept and self-efficacy exert a significant influence on students’ academic behavior, such as performance and motivation. |
| 23 | Fleischmann et al (2021) | 9104 German secondary school students | test | multilevel structural equation model | academic self-concept, achievement | Students usually suffered from lower academic self-concept, in company with higher-achieving classmates than lower-achieving classmates. |
| 24 | Gorges et al. (2018) | 446 German elementary students | scale, test | confirmatory factor analysis | academic self-concept, performance | As for children with learning disabilities, the reciprocal effect between academic self-concept and performance weakens. |
| 25 | Guay et al. (2010) | 925 Canadian high school students | questionnaire, scale | structural equation modeling analysis | academic self-concept, motivation, achievement | There exist mediational and additive effects among academic self-concept, motivation, and achievement. |
| 26 | Guay et al. (2019) | 820 Canadian kindergarten students | survey, interview | structural equation modeling analysis | academic self-concept, motivation, achievement | Academic self-concept positively mediates the relation between intrinsic motivation and achievement. |
| 27 | Guggemos (2021) | 202 Swiss high school students | questionnaire | latent growth curve modeling | academic self-concept, self-efficacy | Academic self-concept, such as computer thinking self-concept was proved to be tightly associated with self-efficacy. |
| 28 | Guo et al. (2020) | 302 Chinese secondary school Miao students | scale, questionnaire | structural equation models | academic self-concept, performance | Academic self-concept is conducive to boost students’ problem solving performance. |
| 29 | Karimova et al. (2020) | 540 Azeri secondary school students | online test, questionnaire | confirmatory factor analysis | academic self-concept, achievement | The correlation between academic self-concept and achievement is domain-specific. |
| 30 | Keller et al. (2021) | 470,804 elementary and secondary school students across 13 countries | test, scale | polynomial and interrupted regression analyses | academic self-concept, achievement | The relationship between achievement and academic self-concept is not always linear. |
| 31 | Khajavy et al. (2018) | 1200 Iranian secondary school students | questionnaire | doubly latent multilevel analysis | academic self-concept, self-efficacy, motivation, achievement | There is a positive relation between achievement and all the motivational constructs, ranging from academic self-concept, self-efficacy, achievement goals to interest. |
| 32 | Khalaila (2015) | 170 Israeli undergraduates | scale, questionnaire | multiple mediator analysis | academic self-concept, achievement, motivation, performance | Academic self-concept is positively associated with achievement. Moreover, test anxiety has negative effect on achievement, which can be moderated by intrinsic motivation. |
| 33 | Korhonen (2014) | 1152 Finnish secondary school students | scale | latent profile analysis | academic self-concept, performance, self-efficacy | Students with low-performance, negative self-concept, and self-efficacy, are more likely to drop out of school. |
| 34 | Lazarides et al. (2021) | 807 German secondary school students | questionnaire | multi-level modelling, cross-level interaction analyses | academic self-concept, achievement, self-efficacy | Students’ self-concept of ability is a significant predictor of achievement emotions, which can be strengthened by improving teaching quality. |
| 35 | Min et al. (2016) | 3 TIMSS datasets | questionnaire | hierarchical linear modelling | academic self-concept, achievement | Cross-cultural modesty bias may make academic self-concept negatively associated with achievement |
| 36 | Paechter et al. (2022) | 314 Australian university students | questionnaire | structural equation model | academic self-concept, motivation, performance, achievement | High academic self-concept and motivation are conducive to enhance students’ performance and achievement emotions, especially in adverse circumstances. |
| 37 | Pinxten et al. (2013) | 2834 Flemish adolescents | questionnaire | structural equation model | academic self-concept, achievement | The reciprocal effect between academic self-concept and achievement changes over time. |
| 38 | Plieninger et al. (2015) | 35015 German high school students | self-report | multilevel modelling | academic self-concept, achievement | In terms of science, males are susceptible to big-fish-little-pond effect. |
| 39 | Preckel et al. (2008) | 362 German secondary school students | questionnaire | correlation and regression analysis | academic self-concept, motivation, achievement | Compared with average-ability students, there exist larger gender differences in gifted students in terms of academic self-concept, motivation, and achievement. |
| 40 | Raymo et al. (2019) | 297 American secondary school students | questionnaire, self-report, scale | factor analysis, analysis of variance (ANOVA) | academic self-concept, performance | Academic self-concept was not capable of mediating the relation between academic performance and test anxiety. |
| 41 | Retelsdorf et al. (2014) | 1508 German secondary school students | tests and questionnaires | structural equation modeling | academic self-concept, achievement | There exist reciprocal effects between academic self-concept and achievement, which is in accord with skill-development hypothesis and self-enhancement hypothesis. |
| 42 | Savolainen, P. A., et al. (2018) | 669 Finnish secondary school students | scale | multilevel growth curve model | academic self-concept, performance | Gender differences seemed to constantly make a difference to students’ academic behaviors. |
| 43 | Scherer (2013) | 459 German high school students | questionnaire, scale | confirmatory factor analysis | academic self-concept, self-efficacy | Students’ academic self-concept and self-efficacy play a significant role in enhancing students’ enjoyment in chemistry. |
| 44 | Schnitzler et al. (2021) | 397 German high school students | questionnaire | latent profile analysis, person-centered analysis | academic self-concept, achievement, performance | The higher academic self-concept, the higher engagement. The higher engagement, the higher achievement. |
| 45 | Steegh et al. (2021) | 1974 German high school students | scale, survey | moderated mediation analysis | academic self-concept, achievement, performance | Gender stereotypes exerted negative influence on female students’ representation in STEM courses and career. |
| 46 | Stocker et al. (2021) | 990 Arabian high school students | self-description questionnaire | confirmatory factor analysis, structural equation modelling | academic self-concept, achievement | There is a positive relation between academic self-concept and achievement within the domain and negative one across domains. |
| 47 | Tomas et al. (2020) | 614 secondary school students in Dominican Republic | scales | structural equation model | self-efficacy, performance, achievement | Self-efficacy has a positive effect on students’ engagement and behavioral engagement usually leads to academic success. |
| 48 | Van de Gaer et al. (2012) | 353403 students from 53 countries | test, questionnaires | multi-level regression model | academic self-concept, achievement | In terms of cross-cultural comparison, academic self-concept may be negatively and paradoxically associated with achievement. |
| 49 | Wirthwein et al. (2020) | 425 German high school students | test | confirmatory factor analysis | academic self-concept, motivation | The influence of gender stereotypes of academic self-concept on motivation may vary from boys to girls |
